# Photoactivatable platinum anticancer complex can generate tryptophan radicals†
†Electronic supplementary information (ESI) available: Experimental procedures and additional results from photoactivation studies. See DOI: 10.1039/c8cc06496b


**DOI:** 10.1039/c8cc06496b

**Published:** 2018-11-23

**Authors:** Claudio Vallotto, Evyenia Shaili, Huayun Shi, Jennifer S. Butler, Christopher J. Wedge, Mark E. Newton, Peter J. Sadler

**Affiliations:** a Department of Physics , University of Warwick , CV4 7AL , Coventry , UK . Email: m.e.newton@warwick.ac.uk; b Department of Chemistry , University of Warwick , CV4 7AL , Coventry , UK . Email: p.j.sadler@warwick.ac.uk; c Department of Chemical Sciences , University of Huddersfield , Queensgate , Huddersfield , HD1 3DH , UK

## Abstract

l-Tryptophan (Trp), melatonin (MLT) and the Trp-peptide pentagastrin quenched the formation of azidyl radicals generated on irradiation of the anticancer complex *trans*,*trans*,*trans*-[Pt(pyridine)_2_(N_3_)_2_(OH)_2_] with visible light, giving rise to C3-centred indole radicals which were characterized for Trp and MLT using an EPR spin-trap; indole, together with azidyl and hydroxyl radicals, have potential roles in a multitargeting mechanism of action against resistant cancers.

## Abstract 2

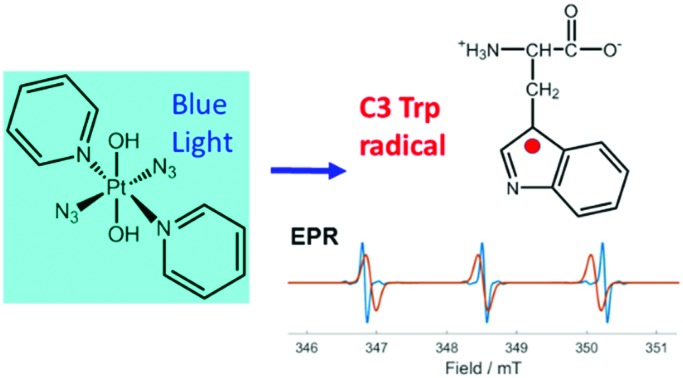
 l-Tryptophan (Trp), melatonin (MLT) and the Trp-peptide pentagastrin quenched the formation of azidyl radicals generated on irradiation of the anticancer complex *trans,trans,trans*-[Pt(pyridine)_2_(N_3_)_2_(OH)_2_] with visible light, giving rise to C3-centred indole radicals which were characterized for Trp and MLT using an EPR spin-trap.

## 

Photo-Activated Chemotherapy (PACT) is an attractive treatment for cancer on account of its potential for selective damage to tumours through the use of spatially directed light whilst causing minimal damage to normal tissues. The selectivity is even higher if the photosensitizer or photoactive prodrug is preferentially taken up by the cancer cells.^1^ Moreover, photoactive compounds can cause unusual damage to cancer cells that is not easily repaired and can therefore be effective against resistant cancers.^2^

Current clinical photosensitizers rely on the conversion of ground-state ^3^O_2_ to reactive excited state ^1^O_2_ to kill cancer cells. Azido Pt(iv) prodrugs have interesting potential for photochemotherapy since they can kill cancer cells *via* mechanisms independent of oxygen, which might be advantageous considering that many tumours are relatively hypoxic. Irradiation of complexes such as *trans*,*trans*,*trans*-[Pt(pyridine)_2_(N_3_)_2_(OH)_2_] (**1**, Fig. 1) with UVA or visible light can lead not only to photoreduction of Pt(iv) to Pt(ii), which can form interstrand cross-links on DNA, but also to the production of azidyl radicals, hydroxyl radicals, and even singlet oxygen.^3^,^4^

**Fig. 1:**
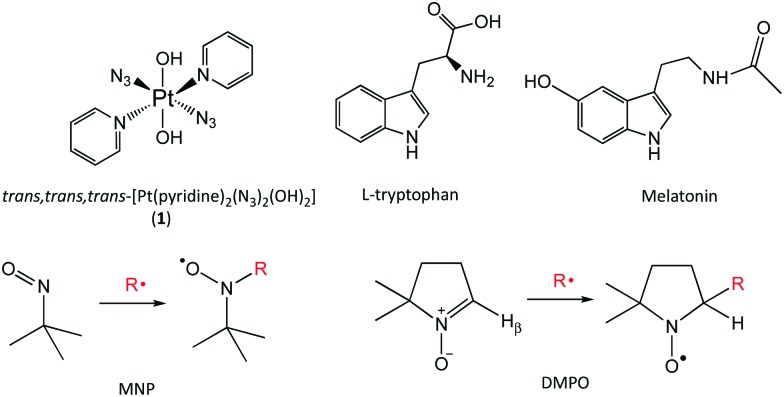
Structures of complex **1**, indole derivatives l-tryptophan and melatonin, spin-trapping agents MNP and DMPO with related radical-spin-trap adduct formation.

Previously we detected azidyl radicals on photoactivation of complex **1** using an EPR spin-trap and observed that trapping of these radicals was quenched by low concentrations of the amino acid l-tryptophan (l-Trp), but not by l-tyrosine.^3^ Recently complex **1** was shown to generate radical oxidation products of l-Trp in a neuropeptide^5^ and an enzyme. Hence the aim^6^ of the present work is to elucidate the nature of the interaction between the azidyl radicals produced by the photodecomposition of complex **1**, and l-Trp and related indole-containing molecules.

First, the photo-activation of complex **1** in the presence of the spin-trapping agent 2-methyl-2-nitrosopropane (MNP) was investigated. Nitroso spin-trapping agents such as MNP are known for their specificity towards the trapping of carbon-centred radicals.^7^ A solution of complex **1** (5 mM) in the presence of an excess of MNP (80 mM) was irradiated in 50 mM phosphate buffer (p.b.), pH 7.2 with a 465 nm blue LED. No EPR signal other than di-*tert*-butyl nitroxide (DTBN), which is expected in spin-trapping experiments,^8^ was observed in the dark. Photo-irradiation of the sample resulted in the progressive increase of the DTBN signal, that is known to be promoted by light^9^ (Fig. S2, ESI†), but, additionally, several low intensity lines were observed (Fig. S3a, ESI†). However, previously reported hyperfine couplings for the MNP-N_3_ spin-adduct^10^ did not match the observed signal (Fig. S3b, ESI†).

When the same solution containing also an excess of l-Trp (40 mM) was irradiated with blue light, an additional 3-line species was detected which overlapped the peaks of DTBN, but was easily distinguished, being broader and characterised by a slightly smaller hyperfine coupling (Fig. S4, ESI†). This signal was attributed to the spin-adduct MNP-Trp, with a neutral l-Trp radical being trapped by the nitroso spin-trapping agent. The EPR parameters extracted by simulation (Table S1, ESI†, Fig. 2) are in good agreement with published values for the tryptophan radical formed by UV photolysis and subsequently trapped with MNP.^11^ Previous EPR studies carried out at low temperatures suggested that photolysis of Trp induced the rupture of the N–H indole bond, and following electron rearrangement the unpaired electron locates in position 3 of the indole ring^12^ (Fig. 3). Indeed, if the unpaired electron were to be located in any other position, additional hyperfine splitting would be detected from the spin-trapped adduct. The detection of such transient species from the photoactivation of complex **1**, here isolated for the first time, suggests that quenching of the azidyl radicals proceeds through a one-electron oxidation with formation of an intermediate tryptophan radical. The concentration of the Trp radical-spin adduct, which was determined by using a calibration curve obtained from standard solutions of 4-hydroxyl-2,2,6,6-tetramethyl-piperidine-1-oxyl (TEMPOL),^13^ was estimated to reach a maximum concentration of 0.7 μM after *ca.* 16 min of irradiation and slowly decreased afterwards (Fig. S5, ESI†). Importantly, the formation of the same Trp spin adduct was observed by performing the experiment in the presence of glutathione (GSH), or by using the cell culture medium RPMI-1640 instead of p.b. to mimic a cell culture environment (Fig. S6 and S7, ESI†)

**Fig. 2:**
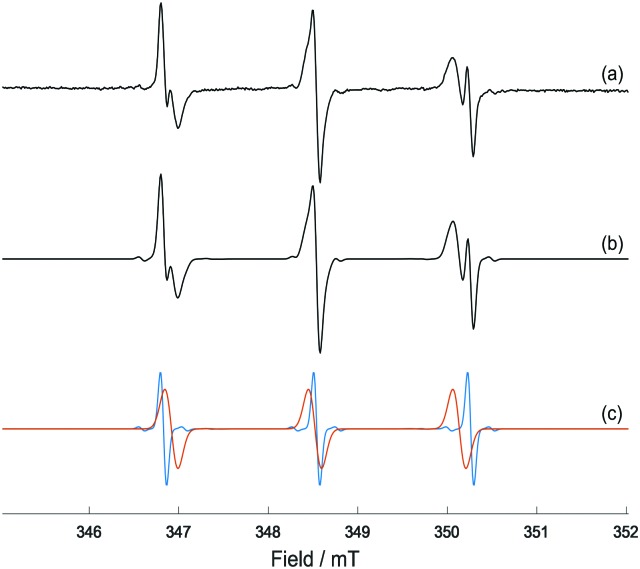
(a) X-band EPR spectrum of MNP-Trp spin adduct formed from photo-irradiation of complex **1** (5 mM), l-Trp (40 mM) and MNP (80 mM) in 50 mM p.b. at pH 7.2 after 41 min of irradiation with 465 nm blue LED light; (b) EasySpin^27^ simulation of the EPR spectrum for a combination of MNP-Trp spin adduct and DTBN; (c) individual simulations of MNP-Trp spin adduct (red) and DTBN (blue). The relative weights of MNP-Trp and DTBN are, respectively, *ca.* 75% and 25%.

**Fig. 3:**
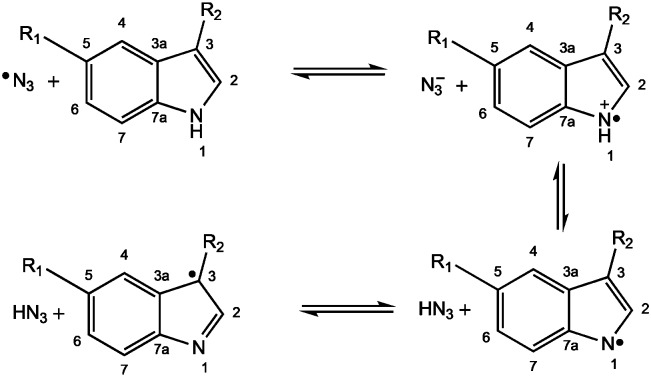
Reaction between the azidyl radical and indole derivatives proceeding through a one-electron transfer pathway and rearrangement to produce the C3-centred indole radical. R_1_ = H (Trp); OCH_3_ (MLT). R_2_ = CH_2_CH(NH_2_)COOH (Trp); CH_2_CH_2_NHCOCH_3_ (MLT).

Photo-activation of complex **1** and the consequent release of azidyl radicals (N_3_˙), trapped by 5,5-dimethyl-1-pyrroline *N*-oxide (DMPO), was previously obtained in low yield with green light (517 nm).^3^ Activation at longer wavelengths is of interest because of their ability to penetrate deeper into tissues, thereby broadening the clinical range of applicability. When a similar solution was irradiated with 525 nm green light, a much weaker 3-line MNP-Trp signal was detected (Fig. S8, ESI† *vs.*Fig. 2) attributable partly to the lower power of the green LED (5.4 mW cm^–2^, *cf.* 7.1 mW cm^–2^ for 465 nm blue LED light).

Next, we investigated the photo-activation of complex **1** in the presence of the indole derivative melatonin. Melatonin (MLT, *N*-acetyl-5-methoxytryptamine, Fig. 1) is a hormone produced by the pineal gland in mammals that regulates the circadian rhythm. Melatonin is an antioxidant towards both reactive oxygen species (ROS) and reactive nitrogen species (RNS), interacting with hydroxyl radicals to produce a melatonin cation radical (MLT˙^+^) which deprotonates at physiological pH to give, similarly to l-Trp, the melatonin neutral radical (MLT˙).^14^ The addition of the hydroxyl radical to the indole ring of melatonin has also been reported.^15^ Solutions containing MLT (40 mM) in 20% EtOH with MNP (80 mM) and complex **1** (5 mM) in p.b. pH 7.2, irradiated with 465 nm light, gave rise to a new, broad 3-line signal in addition to the background DTBN peaks (Fig. S9, ESI†). No EPR signals were detected in the dark, other than DTBN. The EPR parameters of the species extracted by simulation (Fig. S10, ESI†) are in good agreement with those of the MNP-Trp spin adduct (Table S2, ESI†). Therefore, the signal was assigned to the formation of the MNP–MLT spin adduct, with MLT binding to MNP in position 3 of the indole ring, as for l-Trp.

Interestingly, the kinetics of the MNP–MLT and MNP-Trp reactions appeared to differ substantially. Firstly, the concentration of the MNP–MLT adduct (maximum 19.5 μM) was *ca.* 30 times higher compared to MNP-Trp. An explanation for this pronounced difference can be found from the comparison between the reduction potentials of l-Trp (*E*° = 1.015 V)^16^ and MLT (*E*° = 0.73 V).^17^ In fact, although both species possess a reduction potential lower than the azidyl radical (*E*° = 1.33 V),^18^ the much lower reduction potential of MLT suggests that an electron transfer from MLT to the azidyl radical is more favourable than from l-Trp. Secondly, the formation of the melatonin spin adduct appeared to be more sustained, with the maximum reached after 95 min of irradiation *versus* 16 min for l-Trp. Additionally, the curve for formation of the MNP–MLT radical adduct (Fig. 4) suggests the co-participation of two distinct events, a feature not observed with l-Trp. Despite the high concentration of the MNP–MLT adduct formed upon blue light irradiation, no MLT radical signals were detected on irradiation of similar solutions with green light (Fig. S11, ESI†).

**Fig. 4:**
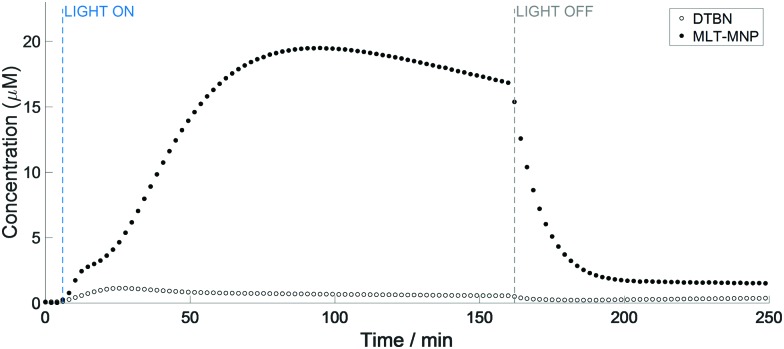
Quantification of the MNP-MLT spin adduct ([black circle]) and DTBN (○) generated from the photo-activation of complex **1** (5 mM) in the presence of MLT (40 mM) and MNP (80 mM) in 50 mM p.b., 20% EtOH, at pH 7.2 with 465 nm LED light.

Although EtOH was used to enhance the solubility of MLT, a repeated experiment in the absence of EtOH gave rise to the same MNP–MLT signals (Fig. S12, ESI†). EtOH is known to be a scavenger specific for the hydroxyl radical (OH˙) forming the α-hydroxyl ethyl radical which can be trapped with both DMPO and MNP, as previously reported.^19^–^21^ When a solution of complex **1** (5 mM) in the presence of 20% EtOH (*ca.* 4 M) and MNP (80 mM) was irradiated with a 465 nm blue LED, a 6-line signal was detected, partially overlapping with the 3-line signal of DTBN (Fig. S13 and S14, ESI†). This species was assigned to the formation of the α-hydroxyl ethyl radical with parameters (Table S3, ESI†) in good agreement with those previously reported for the trapped α-hydroxyl ethyl radical.^20^,^21^ These findings further support the presence of multiple degradation pathways for complex **1**, involving not only the release of azidyl radicals, but hydroxyl radicals too.^5^ The complex kinetics of formation of the melatonin radical can therefore be interpreted as the interaction with both N_3_˙ and OH˙, both contributing to the formation of MLT radicals which are readily trapped by MNP.

l-Histidine (l-His) is also thought to play a role in electron transfer in proteins,^22^ and previous studies reported scavenging properties of l-His towards ROS.^23^,^24^ According to its reduction potential (*E*° = 1.17 V),^25^ histidine is a slightly stronger oxidant than tryptophan, but weaker than the azidyl radical. The reduction potentials therefore suggest that an electron transfer from l-His to azidyl radicals is feasible. However, l-His (100 mM) appeared to have no effect on the products formed from MNP (80 mM) and complex **1** (5 mM) in p.b. pH 7.2 after irradiation with blue light (Fig. S15, ESI†). This may reflect a lower reaction rate to form l-His˙^+^ compared to l-Trp˙^+^ as also observed for radical pair generation from these amino acids.^26^ Rates of l-His radical formation are also expected to be strongly dependent on protonation state so this behaviour may alter with pH.^23^,^26^

To investigate the possible generation of Trp radicals from a peptide, we tested the photo-activation of complex **1** in the presence of pentagastrin: *N*-((1,1-dimethylethoxy)carbonyl)-beta-alanyl-l-tryptophyl-l-methionyl-l-alpha-aspartyl-l-phenylalaninamide (Boc-β-Ala-Trp-Met-Asp-Phe-NH_2_). Pentagastrin is a synthetic analogue of gastrin and can stimulate gastric acid and pepsin secretion. A solution of complex **1** (5 mM) was irradiated with 465 nm light in the presence of pentagastrin (18 mM in DMF 70%) and MNP (80 mM) in 50 mM p.b. pH 7.2. As the photo-irradiation was initiated, the DTBN signal increased with time (Fig. S16, ESI†), together with an additional, low intensity species. The multiplicity of the spin-adduct signal suggested that it originates from the trapping of an N-centred radical. However the same features were detected in the absence of pentagastrin (Fig. S17, ESI†) and were attributed to the solvent, implying that no radicals deriving from pentagastrin as a result of the photo-activation of complex **1** were trapped.

While direct detection of a pentagastrin radical was not possible, the effects of pentagastrin on the trapping of azidyl radicals released upon photo-degradation of complex **1** were examined. A solution of complex **1** (5 mM), DMPO (10 mM) in 50 mM p.b. 70% v/v DMF, at pH 7.2 was irradiated with 465 nm blue LED light in the presence and absence of pentagastrin (20 mM). No signals were detected in the dark. In the absence of pentagastrin, the photo-activation of complex **1** induced the formation of a 12-line signal of pattern 1 : 1 : 1 : 2 : 2 : 2 : 2 : 2 : 2 : 1 : 1 : 1 (Fig. 5), which was assigned to the DMPO-N_3_ spin adduct as previously reported.^3^ The detected splitting arises from the coupling to the nitroxidic nitrogen, the β-proton of the spin trap structure and the α-nitrogen of the azidyl radical, giving 18 lines which partially overlap (Fig. S18 and Table S4, ESI†). The presence of pentagastrin produced a pronounced decrease in the concentration of the DMPO-N_3_ spin adduct, to *ca.* 40% of that detected in the absence of pentagastrin (*ca.* 0.9 μM *vs.* 2.3 μM). The kinetics of formation and degradation of the spin adduct in the two cases (presence and absence of pentagastrin) are reported in Fig. S19 (ESI†). It therefore appears that, even as part of a small peptidic chain, tryptophan preserves its photoprotective effect towards azidyl radicals. The lack of detection of a tryptophan radical intermediate through spin trapping with MNP may be due to the decreased accessibility to the amino acid within the peptidic chain, which could still be available for oxidative attack by N_3_ radicals, but not for trapping with the nitroso spin trap. These findings suggest that azidyl radicals may exert their cytotoxic activity by carrying out oxidative attack on l-Trp residues in proteins, with the consequent disruption of cell homeostasis leading to cell death.

**Fig. 5:**
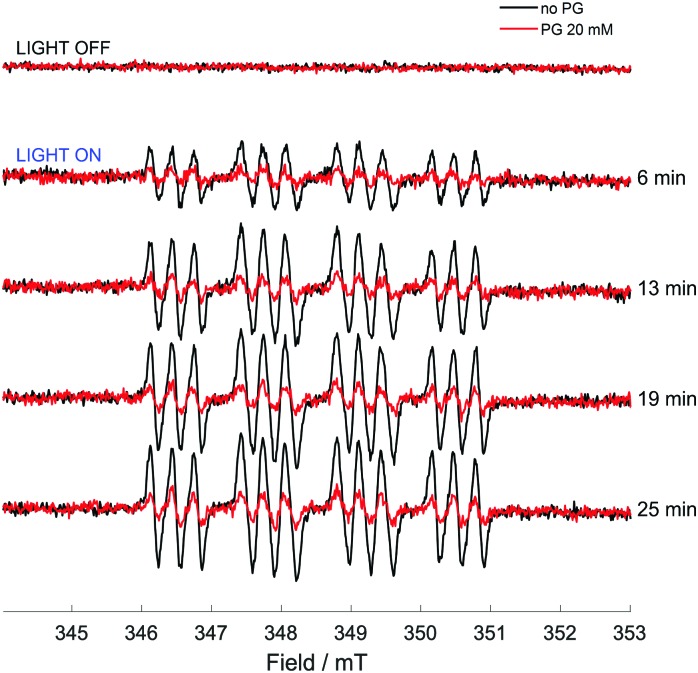
X-band CW EPR spectra of a solution of complex **1** (5 mM), pentagastrin (20 mM) and DMPO (10 mM) in 50 mM p.b., 70% DMF, at pH 7.2, before and during continuous irradiation with a 465 nm blue LED. Each spectrum is the sum of 25 consecutive scans. Times are between the start of irradiation and end of the acquisition of the last scan.

In conclusion, our results reveal key aspects of the mechanism of action of innovative photo-chemotherapeutic platinum(iv) complexes. The photo-activatable platinum(iv) pro-drug *trans*,*trans*,*trans*-[Pt(N_3_)_2_OH_2_(pyridine)_2_] (**1**) appears to have a novel mechanism of anticancer activity involving multitargeted attack on DNA by Pt(ii) photoproducts and attack on other target sites, possibly proteins. The detection of transient l-Trp and MLT radicals by EPR spin trapping experiments, together with the herein reported quenching ability of the tryptophan containing peptide pentagastrin towards RNS, provides strong and direct evidence in this direction. Additionally, the trapping of the α-hydroxy-ethyl radical confirmed the presence of multiple photo-degradation pathways for complex **1**, involving the release of both azidyl and hydroxyl radicals, as summarised in Fig. 6. Such a mechanism of action can help to combat cancer resistance, whilst limiting at the same time unwanted side effects owing to the intrinsic selectivity of photochemotherapy.

**Fig. 6:**
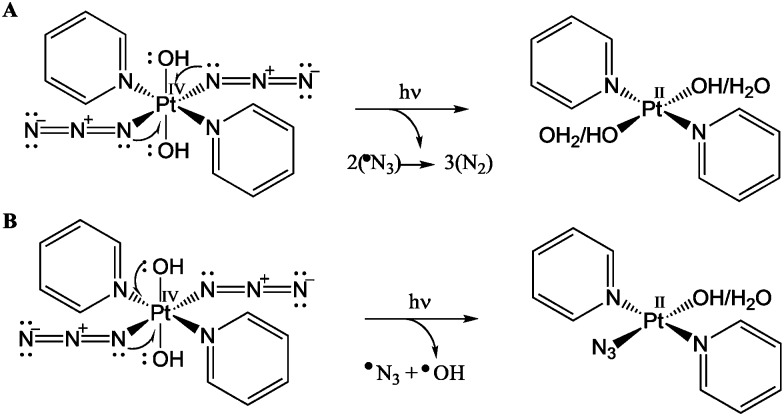
Photo-irradiation of complex **1** leading to the reduction of Pt(iv) to Pt(ii) *via* two one-electron donations from (A) two azides; (B) one azide and one hydroxide ligand.

This work was supported by the EU Marie Curie Initial Training Network FP7-PEOPLE-2012-ITN (Grant no. 316630 CAS-IDP for C. V.), ERC (grant no. 247450), EPSRC (grant no. EP/G006792 and EP/F034210/1), Warwick Chancellor's Scholarship (for H. S.), and Warwick Spectroscopy RTP with the help of Dr Ben Breeze.

## Conflicts of interest

There are no conflicts to declare.

## Supplementary Material

Supplementary information
