# Supplementary material for: Photoactivatable platinum anticancer complex can generate tryptophan radicals
Source: Chem Commun (Camb). 2018 Nov 23;54(98):13845–8. doi: 10.1039/c8cc06496b (PMC6336088; doi:10.1039/c8cc06496b)
Supplement: Supplementary file 1 [file CC-054-C8CC06496B-s001.pdf]

Electronic Supplementary Material (ESI) for Chemical Communications.
This journal is © The Royal Society of Chemistry 2018

Photoactivatable platinum anticancer complex can generate tryptophan radicals

Claudio Vallotto, Evyenia Shaili, Huayun Shi, Jennifer S. Butler, Christopher J. Wedge, Mark E. Newton and Peter J. Sadler

Electronic Supporting Information

Contents

Materials 2
Sample preparation 2
Quantification of spin adducts 2
EPR spectroscopy 2
Irradiation 3
EPR simulations 3
References 22

Supplementary Tables

Table S1 EPR hyperfine couplings and *g*-values for MNP-Trp and DTBN 4
Table S2 EPR hyperfine couplings and *g*-values for MNP-MLT and DTBN 5
Table S3 EPR hyperfine couplings and *g*-values for MNP-EtOH) and DTBN 5
Table S4 EPR hyperfine couplings and *g*-values for DMPO-N_3 6

Supplementary Figures

Fig. S1 Setup of the X-band EPR cavity for the irradiation experiments 3
Fig. S2 Photo-activation of complex **1** in the presence of MNP 7
Fig. S3 Photo-activation of complex **1** in the presence of MNP and simulated MNP-N3 spin adduct 8
Fig. S4 Photo-activation of complex **1** in the presence of MNP and L-Trp 9
Fig. S5 Kinetics of formation of the L-Trp-MLT spin adduct (blue light). 9
Fig. S6 Photo-activation of complex **1** in the presence of MNP, L-Trp and GSH 10
Fig. S7 Photo-activation of complex **1** in the presence of MNP and L-Trp in RPMI-1640 11
Fig. S8 Photo-activation of complex **1** in the presence of MNP and L-Trp (green light) 12
Fig. S9 Photo-activation of complex **1** in the presence of MNP and MLT (20% EtOH) (blue light) 13
Fig. S10 Photo-activation of complex **1** in the presence of MNP and MLT (20% EtOH) (blue light) and simulated MNP-MLT spin adduct 14
Fig. S11 Photo-activation of complex **1** in the presence of MNP and MLT (20% EtOH) (green light) 15
Fig. S12 Photo-activation of complex **1** in the presence of MNP and MLT (H2O) (blue light) 16
Fig. S13 Photo-activation of complex **1** in the presence of MNP and EtOH (blue light) 17
Fig. S14 Photo-activation of complex **1** in the presence of MNP and EtOH and simulated MNP-EtOH spin adduct. 18
Fig. S15 Photo-activation of complex **1** in the presence of MNP and L-His 19
Fig. S16 Photo-activation of complex **1** in the presence of MNP and pentagastrin (blue light) 19
Fig. S17 Photo-activation of complex **1** in the presence of MNP and pentagastrin (summed scans) 20
Fig. S18 Photo-activation of complex **1** in the presence of DMPO 70% (DMF) and simulated DMPO-N_3 spin adduct 21
Fig. S19 Kinetics of formation of the DMPO-N_3 spin adduct in the presence and absence of pentagastrin 22

Materials

Trans,trans,trans-[Pt(N_3)$_2$(OH)$_2$(py)$_2$] (complex **1**) was synthesised as previously reported.[1] L-Tryptophan (L-Trp), melatonin (MLT), L-histidine (L-His), pentagastrin (N-t-Boc-β-Ala-Trp-Met-Asp-Phe amide, PG), acetonitrile (ACN), dimethylformamide (DMF), glutathion (GSH), 5,5-dimethyl-1-pyrroline N-oxide (DMPO) and 2-methyl-2-nitrosopropane (MNP) dimer were all purchased from Sigma Aldrich. RPMI-1640 medium was purchased from Greiner Bio-One GmbH. MLT, pentagastrin, DMPO and MNP dimer were stored at −20 °C. Absolute ethanol (AR grade) was purchased from Fischer Scientific.

Sample preparation

Solutions containing combinations of complex **1** (5 mM), MNP (80 mM), L-Trp (40 mM), L-His (100 mM), MLT (different concentrations) and GSH (1 mM) were prepared either in pH 7.2 phosphate buffer (p.b.) 50 mM or RPMI-1640 medium. MNP (1.6 M) was prepared by dissolution of the dimer in ACN and subsequent dilution 1:20 in water. MLT (200 mM) was either dissolved in EtOH and subsequently diluted 1:5 in water or dissolved directly in water. Samples *ca.* 100 µL were transferred using a plastic syringe with metal needle (Braun Sterican 4665643; 21 G, 120 mm) to a quartz capillary with inner diameter of 1.0 mm and outer diameter of 2.0 mm (Wilmad LabGlass 712-SQ-100M) and sealed with Parafilm®. Sample tubes were then positioned in the EPR cavity so that the sample solution filled the entire length of the cavity. Sample preparation was done under dim controlled lightning conditions and transfer to the EPR spectrometer was in the dark to prevent the photo-activation of complex **1** prior to the beginning of the experiment.

Quantification of spin adducts

The quantification of the spin adducts was performed by using a calibration curve obtained from standard solutions of 4-hydroxyl-2,2,6,6-tetramethyl-piperidine-1-oxyl (TEMPOL), whose concentrations were checked by optical absorption as previously reported.[2] The EPR spectrum of each solution of TEMPOL was acquired. The spectra were baseline corrected and simulated with EasySpin,[3] with double integration performed on the simulated spectra.

EPR spectroscopy

All EPR spectra were recorded on an X-band *Bruker EMX* CW EPR spectrometer at ambient temperature (*ca.* 295 K) using a TM_{110} cavity (ER 4103TM). A 2,2-diphenyl-1-picrylhydrazyl (DPPH) standard was used for calibration of the g-factor. Sweep time was approximately 13 s per scan and modulation depth was set to 0.1 mT.

Irradiation

The LED was inserted at the end of a plastic tube which was clamped to a support. The TM_{110} cavity is equipped with a grid on the front to allow optical access to the sample (ca. 80% transmission). The tube was therefore placed in contact with the grid of the EPR cavity in order to convey all the light into it (Fig. S 1). In this work, the position of the LED was maintained throughout all the irradiation experiments. The LED was connected to a current generator, which was switched on at the beginning of the irradiation. Either a 465 nm blue light (LED465E, Thorlabs, FWHM 25 nm) or a 525 nm green light (LED528E, Thorlabs, FWHM 40 nm) LED were used for irradiating the samples. Under the operating conditions used the radiation power was measured with a power meter and was found to be 7.1 mW cm^{-2} for the 465 nm diode and 5.4 mW cm^{-2} for the 525 nm diode.

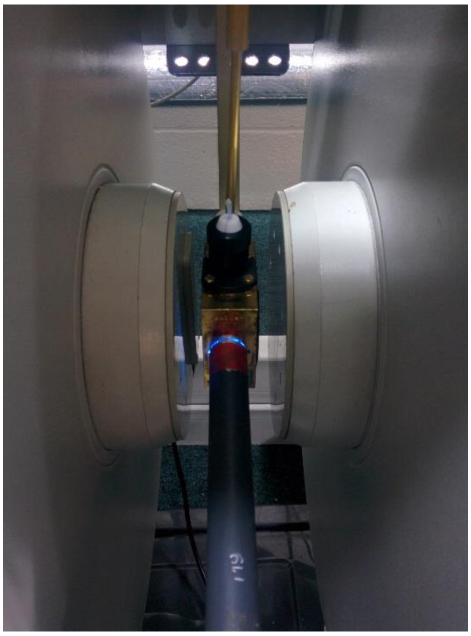

Fig. S1 Setup of the X-band EPR cavity for the irradiation experiments, showing the LED light source inserted at the end of a plastic tube, which is clamped on a support (not shown).

EPR simulations

EPR spectral simulations were performed in Matlab using the EasySpin package.[3] The *garlic* routine (appropriate for the fast-motional regime) was used for all the experiments. Spectral parameters were determined by using EasySpin's *esfit* routine with the Nelder-Mead simplex algorithm.

Simulations of the MNP-tryptophan (MNP-Trp) and the MNP-melatonin (MNP-MLT) spin adducts were performed by including only the hyperfine coupling arising from the nitroxidic nitrogen, which was considered to be fully isotropic. Simulation of the MNP-α-hydroxy-ethyl adduct was performed including the hyperfine couplings of both the nitroxidic nitrogen and

the α-proton. Simulation of the DMPO-N_3 nitrone spin adduct was performed considering couplings to the nitroxidic nitrogen, the β-proton of the spin trapping agent and the α-nitrogen of the trapped azidyl radical.

EPR parameters of the MNP di-adduct di-*tert*-butyl nitroxide (DTBN) were obtained by fitting a spectrum acquired from a solution of MNP which had been illuminated overnight with the 465 nm LED, in order to promote the formation of DTBN. Hyperfine couplings arising from both the nitroxidic nitrogen and statistical abundance of nearest neighbour ^{13}C nuclei were included and considered to be fully isotropic.

An isotropic **g**-tensor was used for all the simulations and dynamic effects were neglected.

Supplementary Tables

Table S1 EPR hyperfine couplings (mT) and *g*-values for the trapped L-Trp radical (MNP-Trp) and the MNP di-adduct di-*tert*-butyl nitroxide (DTBN), comparing the parameters determined in this study with those previously published.

**Radical**		a_{NO}^{N}	$a^{^{13}C}$	**Linewidth (lw_{pp})**	***g*-value**
**MNP-Trp**	This study (465 nm)	1.61(1)	-	0.15	2.0054(2) [a]
	This study (525 nm)	1.62(1)	-	0.16	2.0053(2) [a]
	Ref.[4]	1.63	-	-	- [b]
**DTBN**	This study (465 nm)	1.71(1)	0.43(1)	0.07	2.0053(2) [a]
	Ref.[5]	1.72	-[b]	-[b]	-[b]

[a] determined by comparison with a DPPH standard; [b] not determined.

Table S2 EPR hyperfine couplings (mT) and *g*-values for the trapped MLT radical (MNP-MLT) compared to the MNP-Trp spin adduct and the MNP di-adduct di-*tert*-butyl nitroxide (DTBN).

Radical		a_{NO}^{N}	a^{13C}	Linewidth	*g*-value
**MNP-MLT (20% EtOH)**	This study	1.59(1)	-	0.14(1)	2.0056(2) [a]
**MNP-MLT (H_2O)**	This study	1.61(1)	-	0.12(1)	2.0054(2) [a]
**MNP-Trp**	This study	1.61(1)	-	0.15(1)	2.0054(2) [a]
	Ref.[5]	1.58	-	0.16(1)	- [b]
**DTBN**	This study [c]	1.70(1) [c]	0.51(1) [c]	0.08(1) [c]	2.0054(2) [a,c]
	Ref.[5]	1.72	- [b]	- [b]	- [b]

[a] determined by comparison with a DPPH standard; [b] not determined; [c] determined for the experiment with MLT in EtOH (20%)

Table S3 EPR hyperfine couplings (mT) and *g*-values for the trapped α-hydroxyl ethyl radical (MNP-EtOH) and the MNP di-adduct di-*tert*-butyl nitroxide (DTBN).

Radical		a_{NO}^{N}	a^{13C}	a_{α}^{H}	*g*-value
**MNP-EtOH**	This study	1.56(1)	-	0.18(1)	2.0054(2) [a]
	Ref.[6,7]	1.55	-	0.18	- [b]
**DTBN**	This study	1.71(1)	0.48(1)	-	2.0054(2) [a]
	Ref.[5]	1.72	- [b]	-	- [b]

[a] determined by comparison with a DPPH standard; [b] not determined

Table S4 EPR hyperfine couplings (mT) and *g*-values for the azidyl radical nitrone spin adduct (DMPO-N_3).

**Radical**		a^{N}_{NO}	a^{H}_{β}	$a^{N}_{N\alpha}$	***g*-value**
**DMPO-N_3**	This study (DMF 70%)	1.38(1)	1.30(1)	0.31(1)	2.0055(2) [a]
	Ref.[8] (DMF 50%)	1.38	1.39	0.30	- [b]
	Ref.[9] (H_2O)	1.45	1.49	0.32	- [b]

[a] determined by comparison with a DPPH standard; [b] not determined

Supplementary Figures

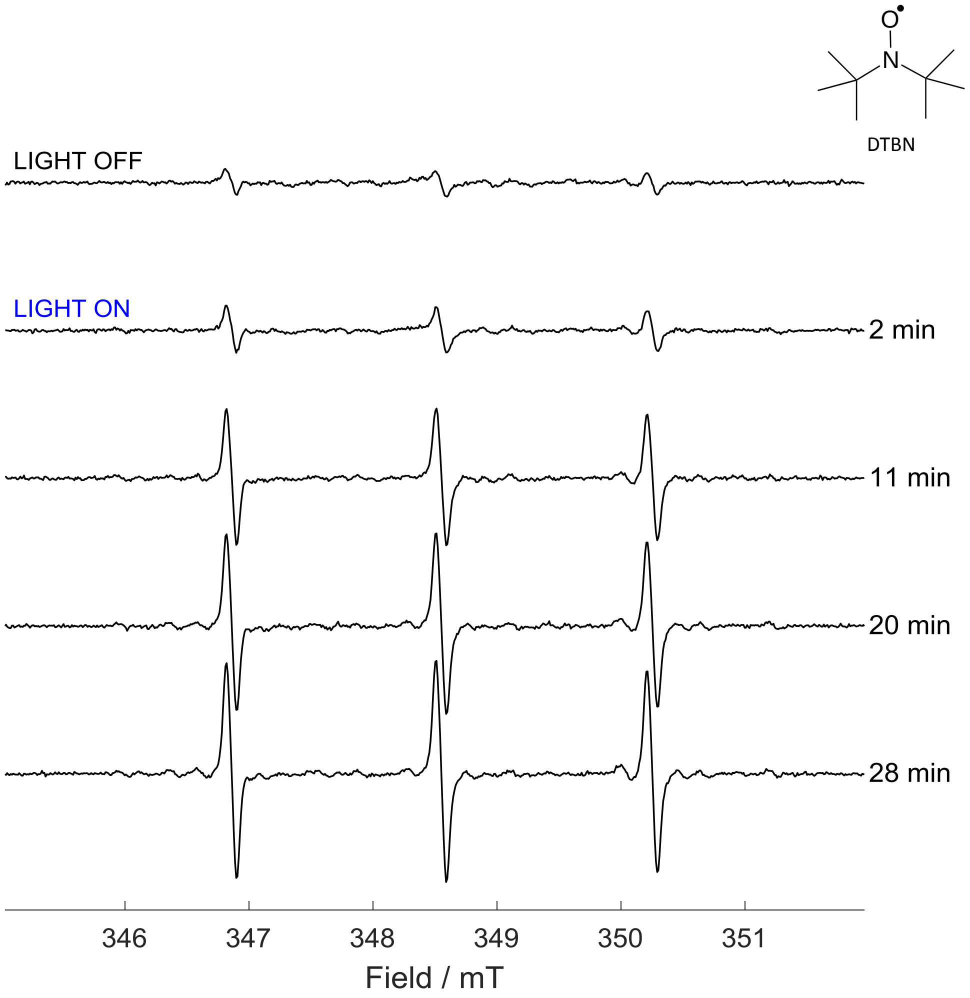

Fig. S2 X-band CW EPR spectrum of a solution of complex **1** (5 mM) and MNP (80 mM) in p.b. 50 mM at pH 7.2, before and during continuous irradiation with a 465 nm blue LED. Each spectrum is the sum of 10 consecutive scans. Times reported refer to the time passed between the start of the irradiation and the end of the acquisition of the last scan included in the spectrum. Top right: structure of di-*tert*-butyl nitroxide (DTBN).

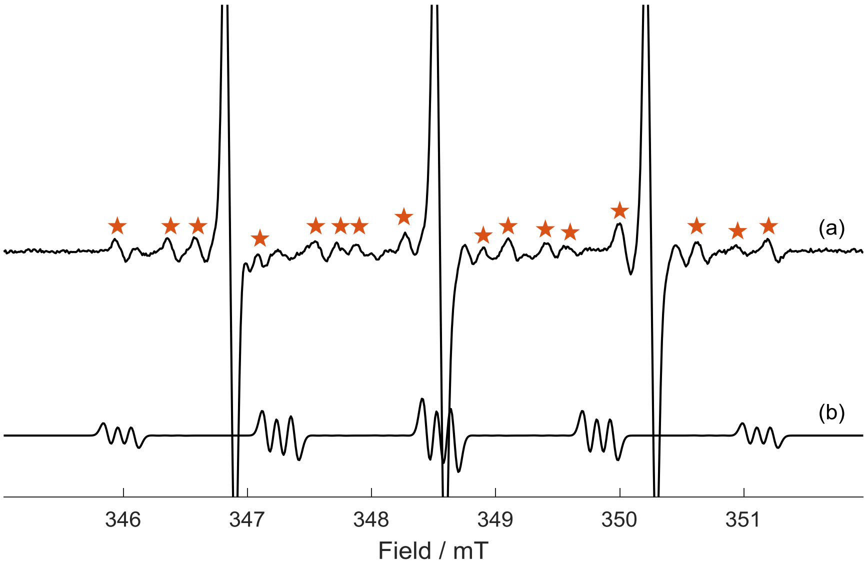

Fig. S3 a) X-band CW EPR spectrum of a solution of complex **1** (5 mM) and MNP (80 mM) in p.b. 50 mM at pH 7.2, after 32 minutes of continuous irradiation with a 465 nm blue LED. The spectrum is the sum of 156 consecutive scans. ★ indicates additional paramagnetic species other than DTBN. b) simulation of the MNP-N_3 spin adduct obtained with previously reported hyperfine couplings ($a_{N1,N2}$ = 1.29 mT; a_{N3} = 0.11 mT).[10]

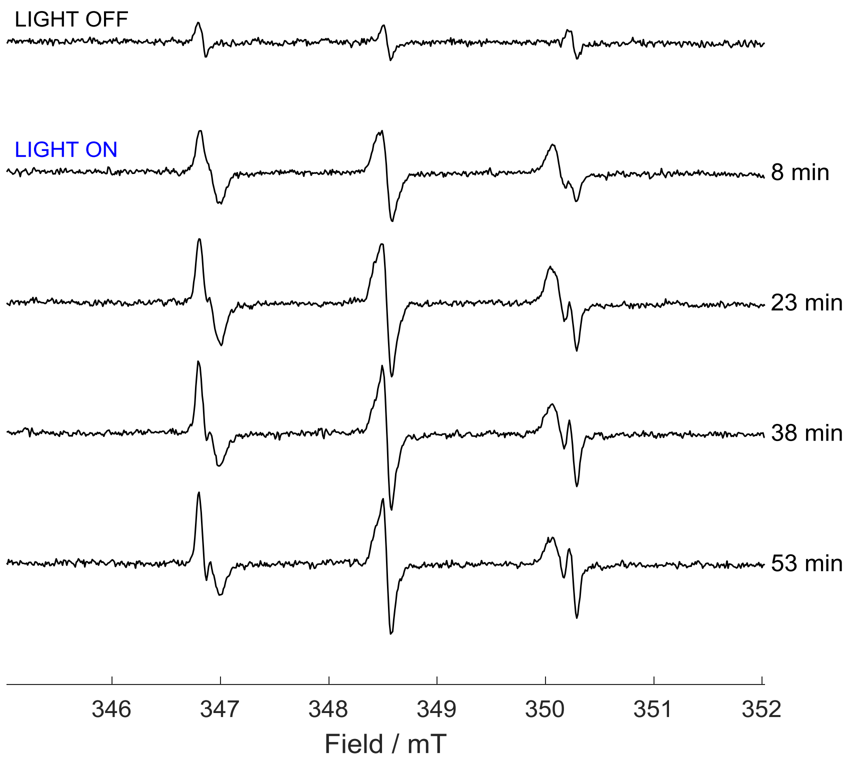

Fig. S4 X-band CW EPR spectrum of a solution of complex 1 (5 mM), L-Trp (40 mM) and MNP (80 mM) in p.b. 50 mM at pH 7.2, before and during continuous irradiation with a 465 nm blue LED. Each spectrum is the sum of 30 consecutive scans. Times reported refer to the time passed between the start of the irradiation and the end of the acquisition of the last scan included in the spectrum.

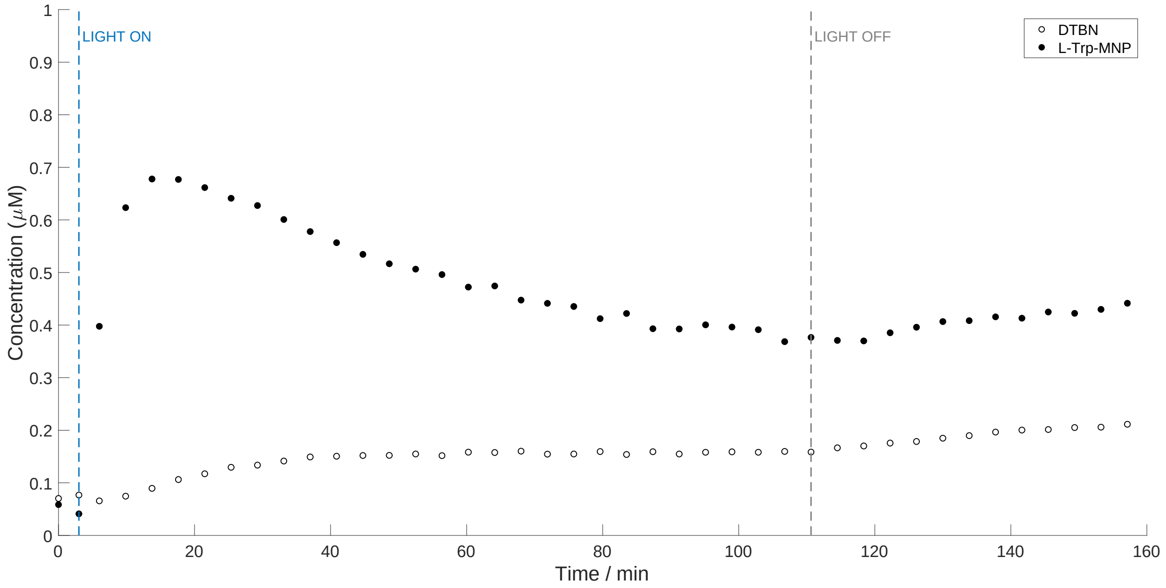

Fig. S5 Quantification of the MNP-Trp spin adduct (●) and DTBN (○) generated from the photo-activation of complex **1** (5 mM) in the presence of L-Trp (40 mM) and MNP (80 mM) with 465 nm LED light prepared in 50 mM p.b. at pH 7.2.

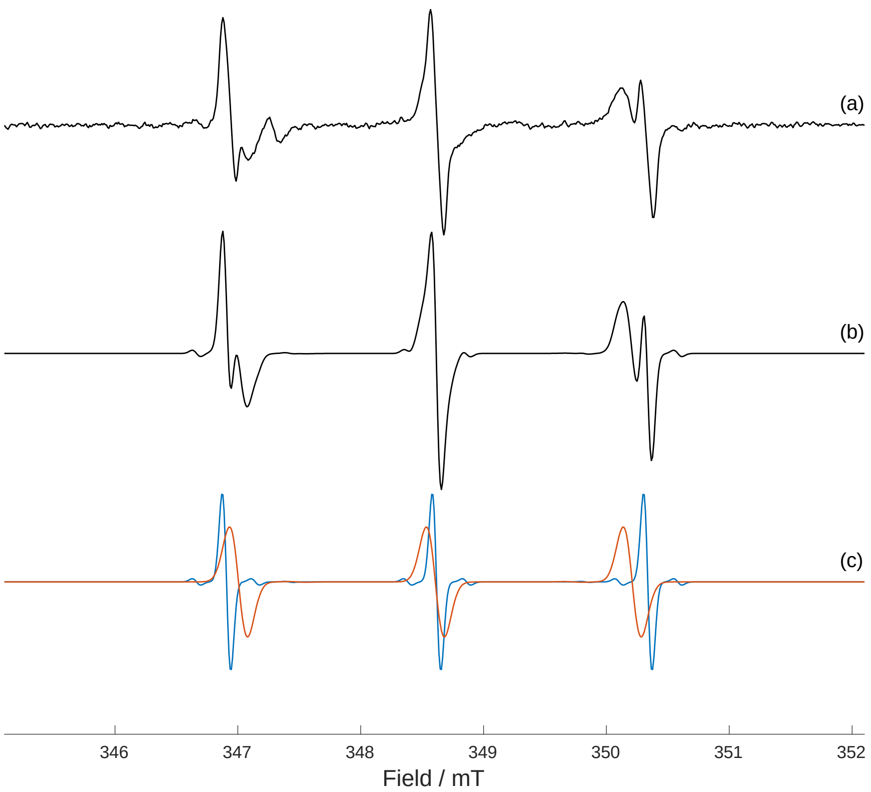

Fig. S6 (a) X-band CW EPR spectrum of a solution of complex 1 (5 mM), L-Trp (40 mM), MNP (80 mM) and GSH (1 mM) in p.b. 50 mM at pH 7.2, during continuous irradiation with a 465 nm blue LED for 8.5 minutes. An experimental field/frequency offset was corrected using the DTBN signal; (b) EasySpin[3] simulation of the EPR spectrum for a combination of MNP-Trp spin adduct and DTBN using hyperfine and linewidth parameters from Table S1 (465 nm); (c) simulation of only the MNP-Trp spin adduct (red) and of only DTBN (blue). The relative weights of MNP-Trp and DTBN are respectively *ca*. 62% and 38%. Trapping of azidyl radicals was obtained under the same experimental conditions by using DMPO as spin trapping agent (data not shown).

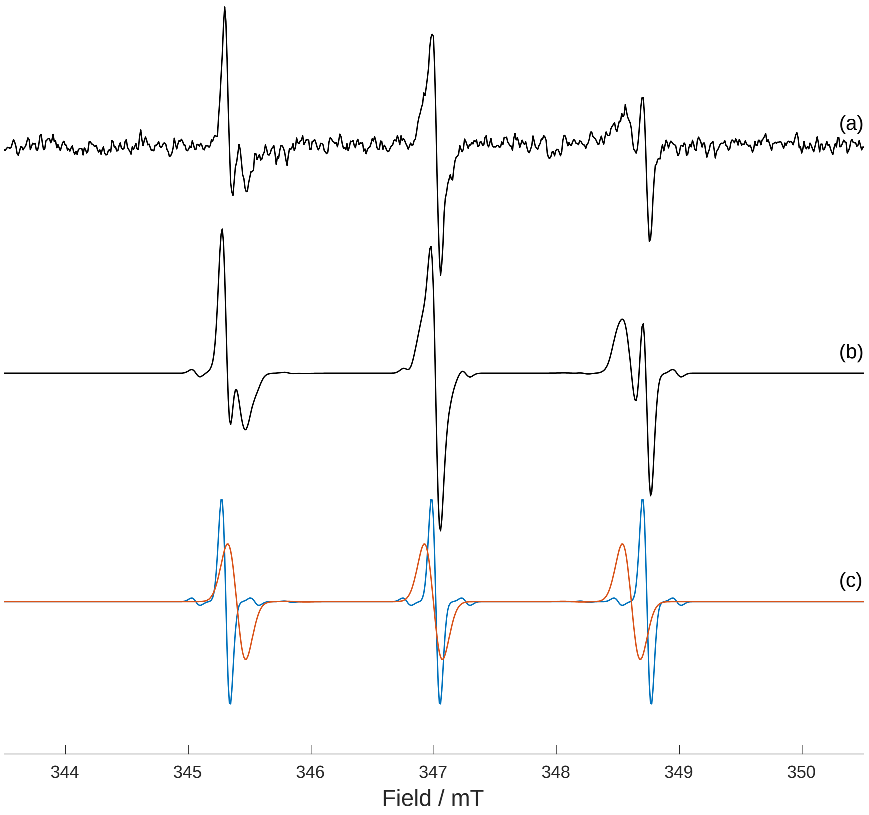

Fig. S7 (a) X-band CW EPR spectrum of a solution of complex 1 (5 mM), L-Trp (40 mM) and MNP (80 mM) in RPMI-1640 medium, during continuous irradiation with a 465 nm blue LED for 8 minutes. An experimental field/frequency offset was corrected using the DTBN signal; (b) EasySpin[3] simulation of the EPR spectrum for a combination of MNP-Trp spin adduct and DTBN using hyperfine and linewidth parameters from Table S1 (465 nm); (c) simulation of only the MNP-Trp spin adduct (red) and of only DTBN (blue). The relative weights of MNP-Trp and DTBN are respectively *ca*. 58% and 42%. Trapping of azidyl radicals was obtained under the same experimental conditions by using DMPO as spin trapping agent (data not shown).

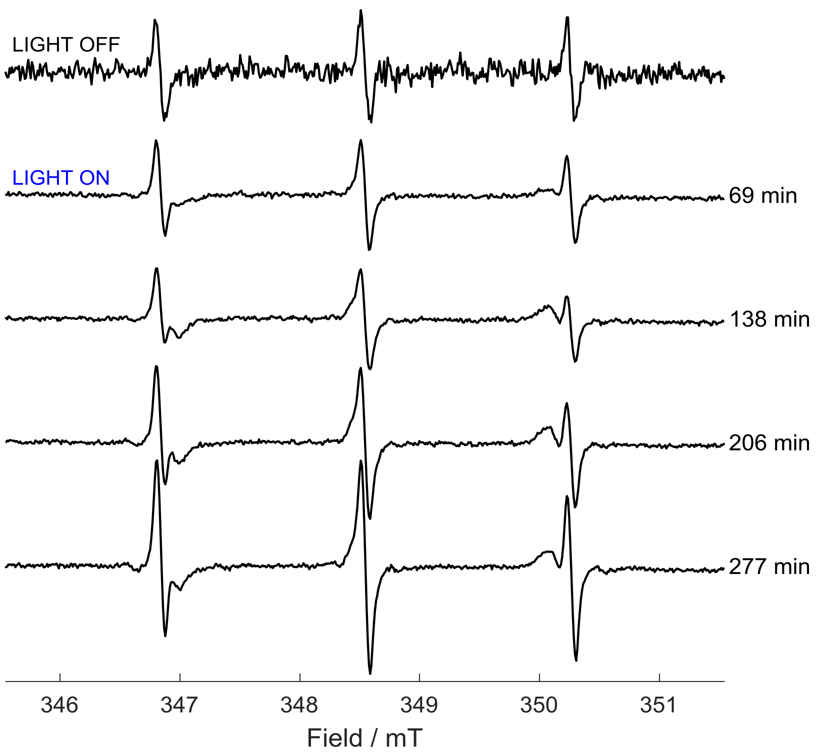

Fig. S8 X-band CW EPR spectrum of a solution of complex **1** (5 mM), L-Trp (40 mM) and MNP (80 mM) in 50 mM p.b. at pH 7.2, before and during continuous irradiation with a 525 nm green LED. Each of the spectra obtained during illumination is the sum of the previous 250 consecutive scans, while the spectrum obtained in the dark is the sum of 10 consecutive spectra and was multiplied by a factor of 25. Times reported refer to the time passed between the start of the irradiation and the end of the acquisition of the last scan included in the spectrum.

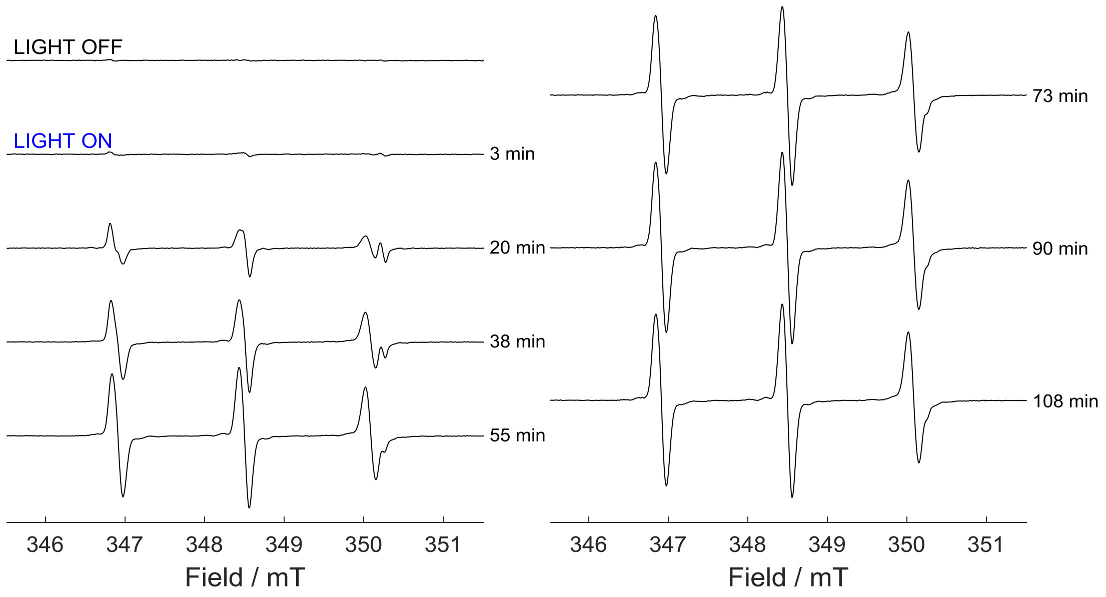

Fig. S9 X-band CW EPR spectrum of a solution of complex **1** (5 mM), MLT (40 mM) and MNP (80 mM) in 50 mM p.b., 20% EtOH at pH 7.2, before and during continuous irradiation with a 465 nm blue LED. Each spectrum is the sum of 10 consecutive scans. Times reported refer to the time passed between the start of the irradiation and the end of the acquisition of the last scan included in the spectrum.

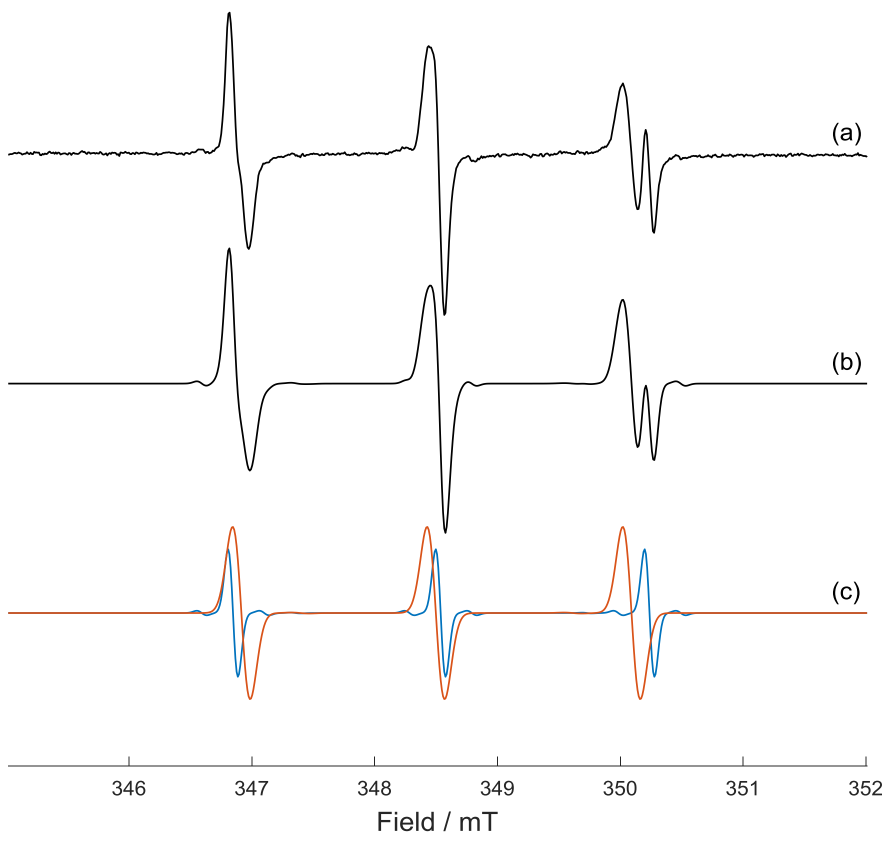

Fig. S10 (a) X-band EPR spectrum of MNP-MLT spin adduct formed from photo-irradiation of complex **1** (5 mM), MLT (40 mM) and MNP (80 mM) in 50 mM p.b., 20% EtOH at pH 7.2 after 51 min of irradiation with 465 nm blue LED light; (b) EasySpin[3] simulation of the EPR spectrum for a combination of MNP-MLT spin adduct and DTBN; (c) simulation of only the MNP-MLT spin adduct (red) and of only DTBN (blue). The relative weights of MNP-MLT and DTBN are respectively *ca*. 80% and 20%.

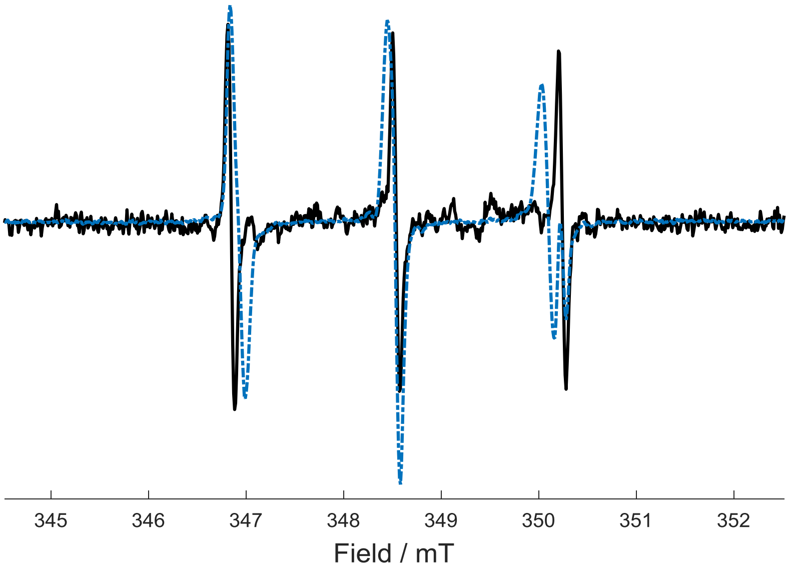

Fig. S11 X-band CW EPR spectrum of a solution of complex **1** (5 mM), MLT (40 mM) and MNP (80 mM) in 50 mM p.b., 20% EtOH at pH 7.2 and irradiated with a 525 nm green LED (black) in comparison to irradiation with 465 nm blue LED (dashed dotted blue). The spectrum obtained with green light irradiation is the sum of 200 scans, while the spectrum obtained with blue light irradiation is the sum of 8 scans.

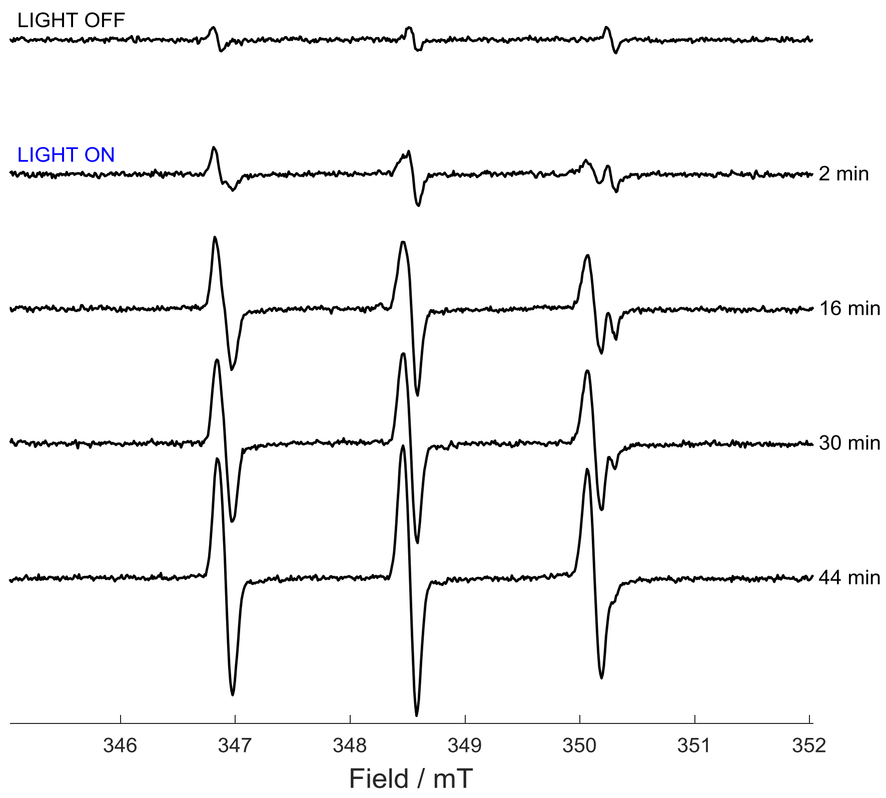

Fig. S12 X-band CW EPR spectra of a solution of complex **1** (5 mM), MLT (0.4 mM) and MNP (80 mM) in 50 mM p.b., at pH 7.2, before and during continuous irradiation with a 465 nm blue LED. Each spectrum is the sum of 10 consecutive scans. Times reported refer to the time passed between the start of the irradiation and the end of the acquisition of the last scan included in the spectrum.

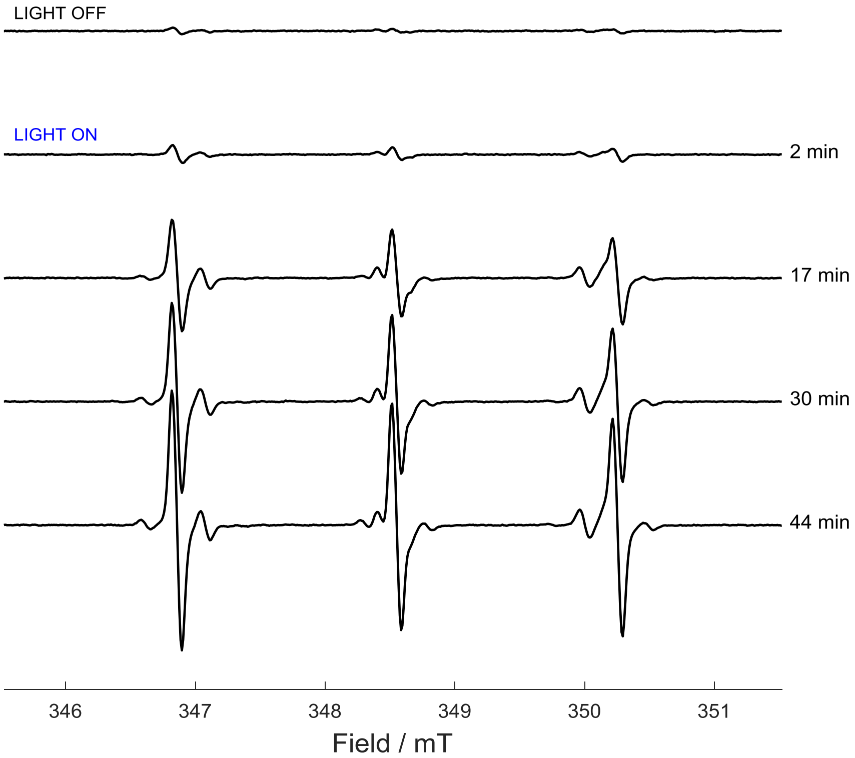

Fig. S13 X-band CW EPR spectra of a solution of complex **1** (5 mM) in the presence of 20% EtOH and MNP (80 mM) in 50 mM p.b., at pH 7.2, before and during continuous irradiation with a 465 nm blue LED. Each spectrum is the sum of 10 consecutive scans. Times reported refer to the time passed between the start of the irradiation and the end of the acquisition of the last scan included in the spectrum.

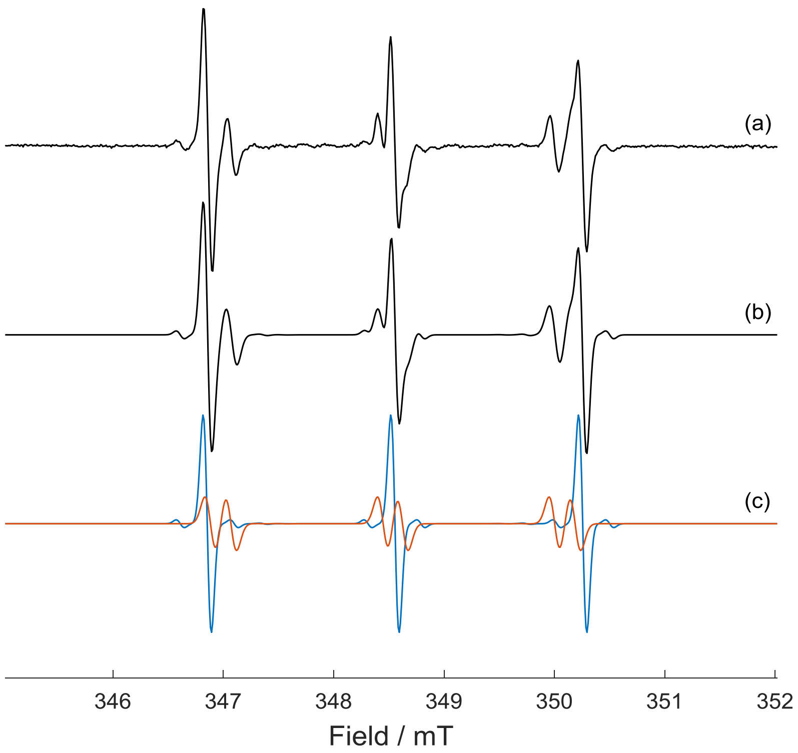

Fig. S14 (a) X-band EPR spectrum obtained from photo-irradiation of complex **1** (5 mM), in the presence of EtOH 20% and MNP (80 mM) in 50 mM p.b., at pH 7.2 after 10 min of irradiation with 465 nm blue LED light; (b) EasySpin[3] simulation of the EPR spectrum for a combination of MNP-EtOH spin adduct and DTBN; (c) simulation of only MNP-EtOH spin adduct (red) and of only DTBN (blue). The relative weights of MNP-EtOH and DTBN are, respectively, *ca*. 46% and 54%.

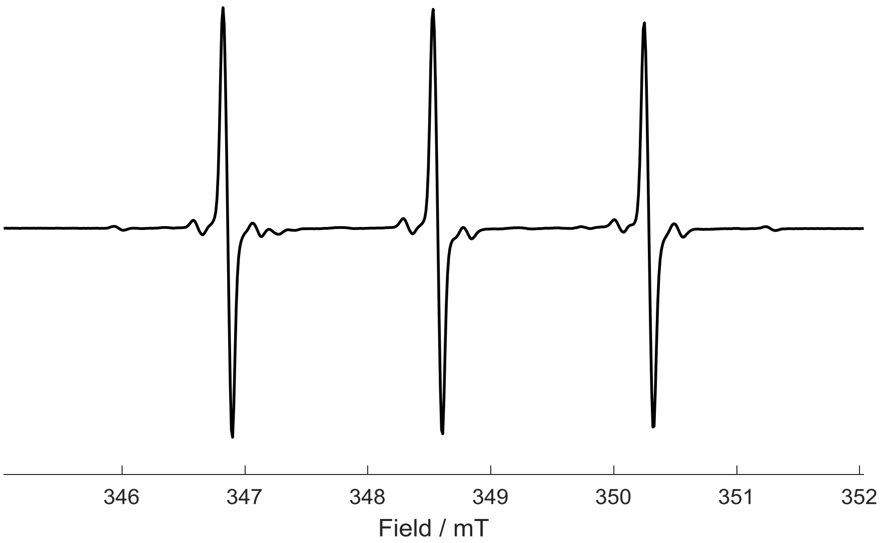

Fig. S15 X-band EPR spectrum obtained from photo-irradiation of complex 1 (5 mM), in the presence of L-His (100 mM) and MNP (80 mM) in 50 mM p.b., at pH 7.2 after 65 min of irradiation with 465 nm blue LED light.

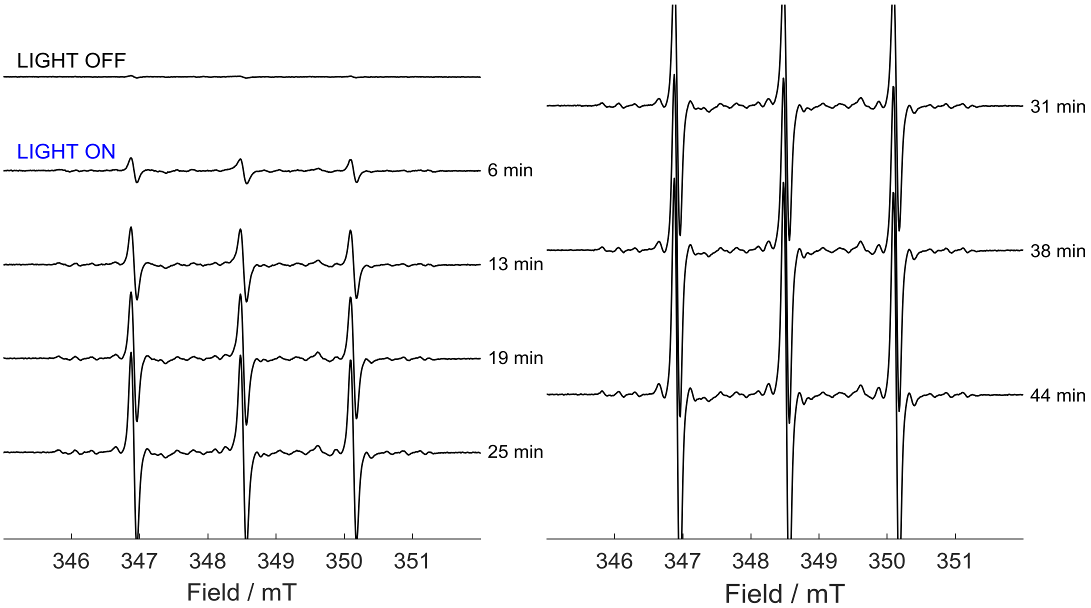

Fig. S16 X-band CW EPR spectra of a solution of complex **1** (5 mM), pentagastrin (18 mM in DMF 70%) and MNP (80 mM) in 50 mM phosphate buffer, at pH 7.2, before and during continuous irradiation with a 465 nm blue LED. Each spectrum is the sum of 25 consecutive scans. Times reported refer to the time passed between the start of the irradiation and the end of the acquisition of the last scan included in the spectrum.

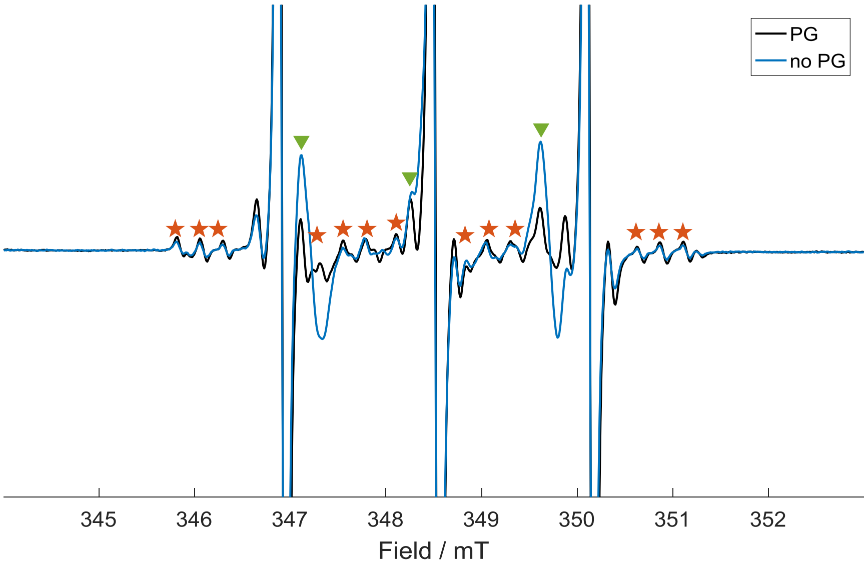

Fig. S17 X-band CW EPR spectra of a solution of complex **1** (5 mM), and MNP (80 mM) in 50 mM p.b., 70% DMF at pH 7.2 in the presence (black) and absence (blue) of pentagastrin (18 mM) after 75 min continuous irradiation with a 465 nm blue LED. Each spectrum is the sum of 300 consecutive scans. ★ and ▼ indicate additional paramagnetic species formed upon irradiation.

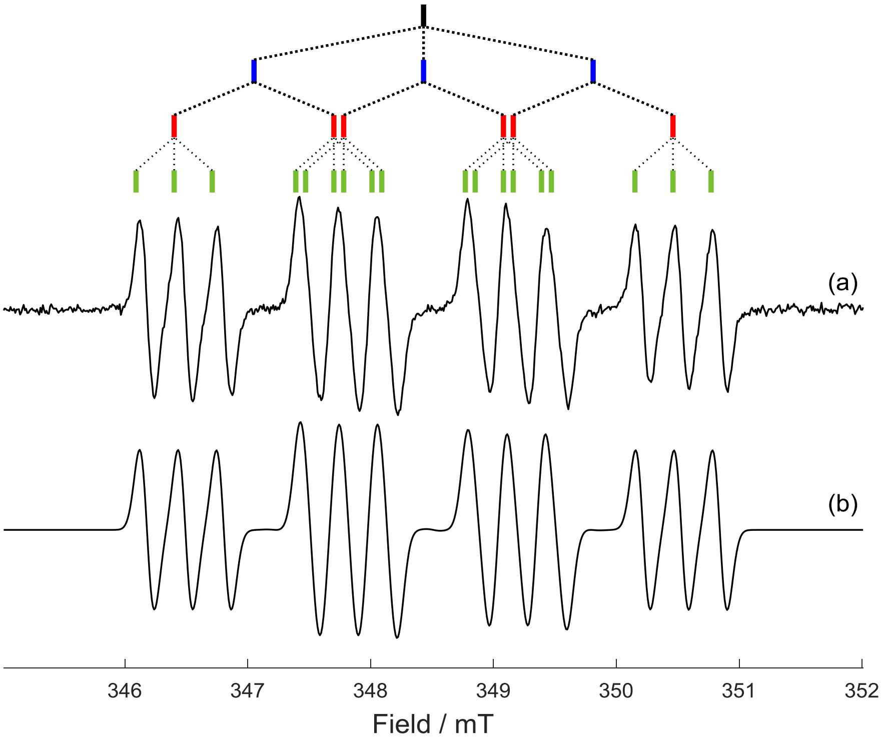

Fig. S18 (a) X-band EPR spectrum obtained from photo-irradiation of complex **1** (5 mM) in the presence of DMPO (10 mM) prepared in 50 mM p.b., 70% DMF at pH 7.2, after 43 min of irradiation with 465 nm blue LED light; the spectrum is the sum of 70 slices; (b) EasySpin[3] simulation of the EPR spectrum with the parameters reported in Table S4. The line diagram for the DMPO-N3 spin adduct is also shown.

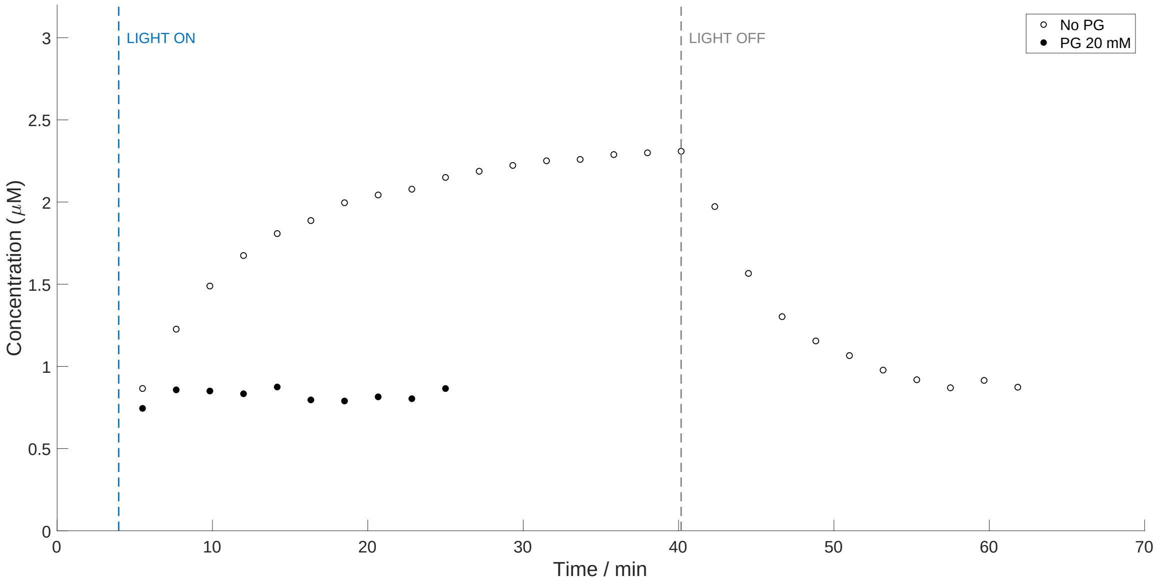

Fig. S19 Quantification of the DMPO-N_3 spin adduct in the presence (●) and absence (○) of pentagastrin (20 mM) generated from the photo-activation of complex **1** (5 mM) in the presence of DMPO (10 mM) in 50 mM p.b., 70% DMF at pH 7.2 with 465 nm LED light.

References

1 J. S. Butler, PhD Thesis, University of Warwick, 2014.

2 D. Barr, J. Jiang and R. T. Weber, *How to Quantitate Nitroxide Spin Adducts Using TEMPOL*, Bruker EPR Experimental Techniques Note 3, Bruker Instruments Inc., Billerica, MA, n.d.

3 S. Stoll and A. Schweiger, *J. Magn. Reson.*, 2006, **178**, 42–55.

4 M. M. Mossoba, K. Makino and P. Riesz, *J. Phys. Chem.*, 1982, **86**, 3478–3483.

5 Y. Lion, M. Kuwabara and P. Riesz, *J. Photochem. Photobiol.*, 1982, **35**, 43–52.

6 G. R. Buettner, *Free Radic. Bio. Med.*, 1987, **3**, 259–303.

7 R. J. Elias, M. L. Andersen, L. H. Skibsted and A. L. Waterhouse, *Am. J. Enol. Vitic.*, 2009, **60**, 471–476.

8 K. Reszka, P. Kolodziejczyk. and J. W. Lown, *Free Radic. Biol. Med.*, 1988, **5**, 63–70.

9 J. S. Butler, J. A. Woods, N. J. Farrer, M. E. Newton and P. J. Sadler, *J. Am. Chem. Soc.*, 2012, **134**, 16508–16511.

10 W. Kremers, G. W. Koroll and A. Singh, *Can. J. Chem.*, 1982, **60**, 1597.
